# The sensitivity of radiobiological models in carbon ion radiotherapy (CIRT) and its consequences on the clinical treatment plan: Differences between LEM and MKM models

**DOI:** 10.1002/acm2.14321

**Published:** 2024-03-04

**Authors:** Joanna Góra, Sarah Grosshagauer, Piero Fossati, Marta Mumot, Markus Stock, Mansure Schafasand, Antonio Carlino

**Affiliations:** ^1^ MedAustron Ion Therapy Center Wiener Neustadt Austria; ^2^ Technical University of Vienna Wien Austria; ^3^ Karl Landsteiner University of Health Sciences Krems an der Donau Austria; ^4^ Medical University of Vienna Wien Austria

**Keywords:** carbon ion radiotherapy, LEM/MKM translation, RBE models, relative biological effectiveness, treatment planning strategies

## Abstract

**Purpose:**

Carbon ion radiotherapy (CIRT) relies on relative biological effectiveness (RBE)‐weighted dose calculations. Japanese clinics predominantly use the microdosimetric kinetic model (MKM), while European centers utilize the local effect model (LEM). Despite both models estimating RBE‐distributions in tissue, their physical and mathematical assumptions differ, leading to significant disparities in RBE‐weighted doses. Several European clinics adopted Japanese treatment schedules, necessitating adjustments in dose prescriptions and organ at risk (OAR) constraints. In the context of these two clinically used standards for RBE‐weighted dose estimation, the objective of this study was to highlight specific scenarios for which the translations between models diverge, as shortcomings between them can influence clinical decisions.

**Methods:**

Our aim was to discuss planning strategies minimizing those discrepancies, ultimately striving for more accurate and robust treatments. Evaluations were conducted in a virtual water phantom and patient CT‐geometry, optimizing LEM RBE‐weighted dose first and recomputing MKM thereafter. Dose‐averaged linear energy transfer (LETd) distributions were also assessed.

**Results:**

Results demonstrate how various parameters influence LEM/MKM translation. Similar LEM‐dose distributions lead to markedly different MKM‐dose distributions and variations in LETd. Generally, a homogeneous LEM RBE‐weighted dose aligns with lower MKM values in most of the target volume. Nevertheless, paradoxical MKM hotspots may emerge (at the end of the range), potentially influencing clinical outcomes. Therefore, translation between models requires great caution.

**Conclusions:**

Understanding the relationship between these two clinical standards enables combining European and Japanese based experiences. The implementation of optimal planning strategies ensures the safety and acceptability of the clinical plan for both models and therefore enhances plan robustness from the RBE‐weighted dose and LETd distribution point of view. This study emphasizes the importance of optimal planning strategies and the need for comprehensive CIRT plan quality assessment tools. In situations where simultaneous LEM and MKM computation capabilities are lacking, it can provide guidance in plan design, ultimately contributing to enhanced CIRT outcomes.

## 1 INTRODUCTION

Currently, two radiobiological models are used to calculate RBE‐weighted dose for carbon ion beam radiotherapy (CIRT) in clinical routine. Historically, CIRT was established in Japan (1994), where the mixed beam model was implemented to calculate RBE‐weighted dose.^1^ Further advances and progression of CIRT to active scanning beam delivery required adaptations of that model. It resulted in the implementation of the microdosimetric kinetic model (MKM), originally developed by Hawkins^2^, ^3^, ^4^ and later modified by Inaniwa^5^ for clinical implementation of CIRT at the National Institute of Radiological Sciences (NIRS)—now National Institutes for Quantum Sciences and Technology (QST), Chiba (Japan).^6^ The first European experience with CIRT followed a few years later (1997) at the Gesellschaft für Schwerionenforschung (GSI), Darmstadt (Germany) and since 2008 patients have been treated with CIRT at the facility in Heidelberg (Germany).^7^ European facilities mostly use the local effect model (LEM).^8^, ^9^ Over the years, both biological models underwent certain adaptations; nevertheless, nearly 30 years of clinical experience has been gathered so far, which serves as the golden standard for currently operating and future carbon ion centers in terms of prescribed doses, fractionation schemes, and dose constraints to organs at risk (OARs). Although both models aim at predicting RBE distributions within tissues, they apply different theoretical concepts and assumptions; therefore, their derived, respective RBE‐weighted dose distributions vary significantly and cannot be compared easily. A brief overview of the two models can be found in the materials and methods section.

There were some attempts to translate between MKM and LEM schedules by adjusting the prescribed doses and OAR dose constraints.^10^, ^11^, ^12^, ^13^ It has been proven that MKM generally estimates lower RBE as compared to LEM in certain dose ranges. Molinelli et al.^10^ showed that the MKM median dose, prescribed to the target volume was 5%−15% lower than for LEM (dose range between 3.6 and 4.6 Gy RBE (MKM)). This resulted in adjustments in nominal prescription doses in LEM, to cope with differences in RBE modeling. Furthermore, several groups investigated the effect of both RBE models on the OAR dose constraints for different tumor sites.^10^, ^11^, ^12^, ^13^, ^14^, ^15^, ^16^, ^17^, ^18^ For example, our in‐house validated constraints for brainstem in LEM model are D0.01cc = 46 Gy RBE, D0.7cc = 38 Gy RBE, which would correspond to 36.8 Gy RBE and 29.3 Gy RBE MKM doses, respectively. For the optic system, on the other hand, higher LEM doses than 50 Gy RBE (D1%) and 40 Gy RBE (D20%) would not be allowed, which translates to 40.6 Gy RBE and 29 Gy RBE in MKM, respectively.^13^

Currently, at MedAustron, the RBE weighted dose optimization is based purely on LEM. Until May 2023, more than 400 patients received treatment with CIRT at MedAustron. In many indications (e.g., H&N tumors, sarcoma, and rectal cancer), the Japanese fractionation schemes are used. Dose prescription and OAR constraints are based on clinical experience obtained with the MKM model, but their numerical values have been adapted to account for the difference between the models.^19^ Such adaptations that translate MKM doses into LEM ones are a good estimate but nevertheless they need to be used with caution.

In the context of these two clinically used standards for RBE‐weighted dose evaluation, wherein shortcomings between them can influence clinical decisions, the objective of this study was to highlight specific scenarios for which the translations diverge. Our aim was to propose planning strategies minimizing those discrepancies, with the goal of producing more accurate and robust treatment plans that would be safe and acceptable for both models.

Given that not every commercial treatment planning system (TPS) has the capability of computing both LEM and MKM RBE‐weighted doses, provided evaluations can serve as a guidance in a more efficient and effective treatment planning process. Considering properties of both models in driving clinical decisions will allow for a more conscious and safer treatment, ultimately contributing to overall improvement in CIRT outcomes. To our knowledge, this is the first study focusing on simultaneous use of both models in a clinical setting.

## 2 MATERIALS AND METHODS

### 2.1 RBE models

In CIRT RBE‐weighted dose is prescribed, optimized, and reported according to the ICRU Report 93.^20^ RBE calculation is complex and depends on many physical and biological parameters, such as the detailed description of the mixed field of particles in each voxel (described by LET spectra or by lineal energy spectra), the dose per fraction, the selected endpoint, and the assumptions about the photons dose‐response curve.^1^, ^2^, ^3^, ^4^, ^5^, ^6^, ^7^, ^8^, ^9^

The characteristics of the mixed field of particles depend on the patient anatomy, the number and orientation of beams applied, the use of specific constraints, or even spot distribution.

Theoretically, different photon dose‐response curves can be used for different endpoints and tissues; however, in the vast majority of cases, this is not done.

Over the years, both models have undergone several adaptations to make the RBE‐weighted dose prediction more accurate.^5^, ^21^, ^22^, ^23^, ^24^, ^25^ For this study, only the clinically implemented standards are of interest, namely, LEM I and the latest MKM adaptation made by Inaniwa in 2015.^21^ For simplicity, we will refer to them throughout the text as LEM and MKM.

Table 1 provides the most significant differences between LEM and MKM.

**TABLE 1:** Basic differences between RBE models.

	LEM—HIT approach	MKM—NIRS approach
Theoretical concept	Biological effects depend only on local dose deposition, independent of radiation quality	Specific energy determines the biological effects (derived with Monte Carlo calculation)
Biological endpoint	Cell survival corresponding to late effects in central nervous system (idealized chordoma cell line)	Cell survival corresponding to acute response of tumor cells (HSG cells)
Reference radiation	Photons	Carbon ion beam (350 MeV/u) at center of SOBP with 60 mm width
Adaptations for clinical RBE	Clinical α/β ratio for photons	Rescaling to match previous experiences with mixed beam model. Clinical weighting factor of 2.41
Calculation parameters	α_x _= 0.1 Gy^‐1^, β_x _= 0.05 Gy^−2^, D_t _= 30 Gy, r_n _= 5 µm^a^	α_0 _= 0.172 Gy^−1^, β = 0.0615 Gy^−2^, α_r _= 0.764 Gy^−1^, r_d _= 0.32 µm, r_n _= 3.9 µm^b^
Dose dependence	RBE is dose dependent Dose/fx influences estimation of RBE	RBE almost independent of dose^c^
LET dependence	Idea to predict effects of high‐LET radiation based on the known effects of low‐LET radiation^a^	LET dependent α, saturation correction for high LET

^a^ Explanation of the parameters can be found in reference 9.

^b^ Explanation of the parameters can be found in reference 21.

^c^ The clinical use of MKM, while accounting for the change in shape of the SOBP with dose per fraction is still not accounting for the change in mean RBE with dose per fraction, to be consistent with the previous clinical data. Formally, the model in clinical use considered the absorbed dose in the middle of the passive scattered CIRT SOBP as a reference 21.

^d^ It was reported that LEM I overestimate the RBE in the entrance region (low LET) and underestimate the RBE in the SOBP (high LET).^26^, ^27^

### 2.2 Datasets and dose computation

**FIGURE 1:**
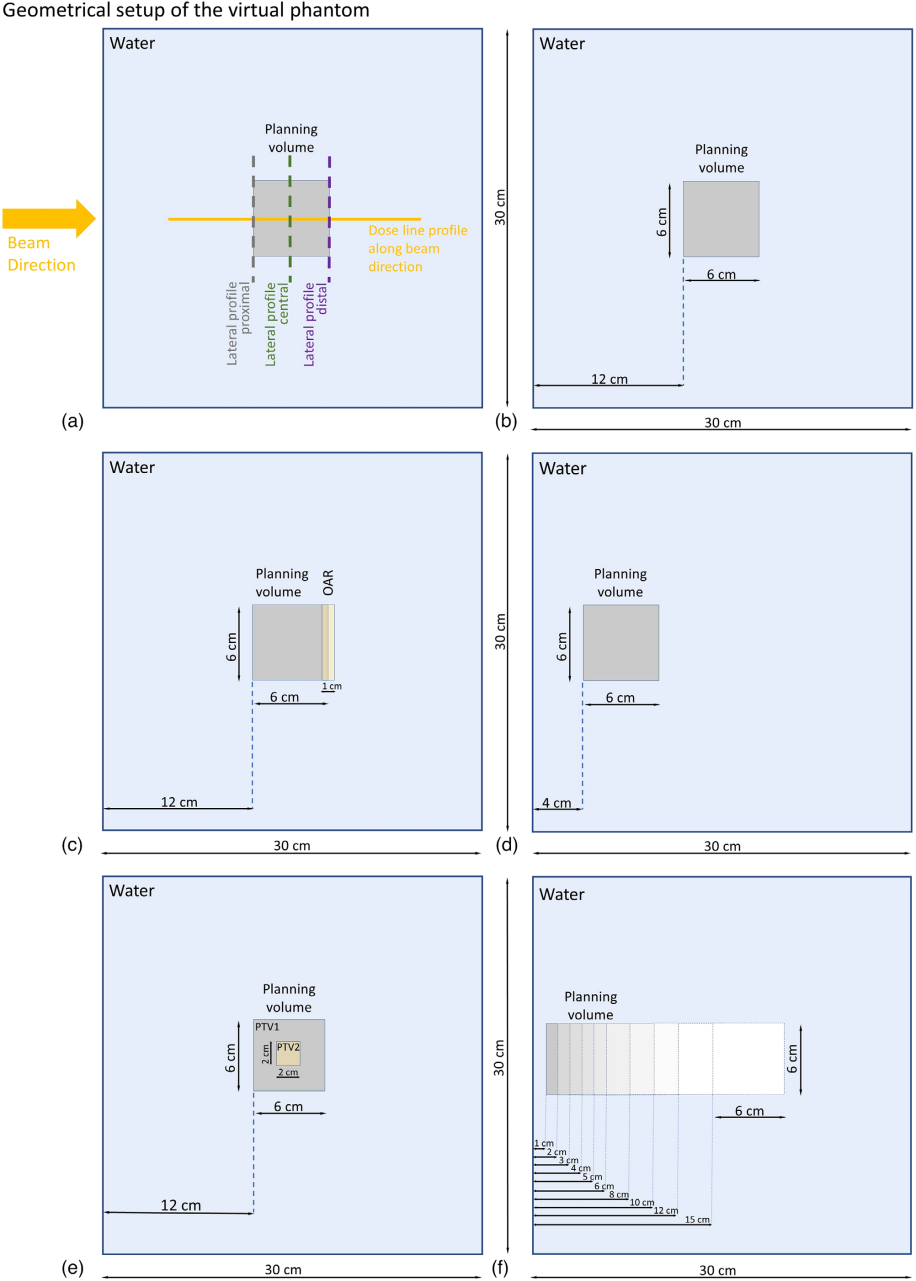
Virtual phantom setup with various geometries (entirely overwritten with water density) used throughout the study. (a) Visualization of selected dose lines for evaluation: depth profile (yellow line), lateral profiles (proximal part of the target (grey), central SOBP region (green), distal part of the target (purple)). (b) General setup used for characteristic of model dependencies (Figure 2, Fig. S.1, Fig. S.2.1). (c) Specific geometry applied for gradient influence considerations (Figure 4). (d) Specific geometry used in considerations related to influence of Rashi (Range shifter) (Fig. S.3.1). (e) Specific geometry used for SIB (Simultaneously Integrated Boost) versus SEB (Sequential Boost) treatment strategy (Fig. S.4). (f) Specific geometry with the planning target volume placed at different depths 1, 2, 3, 4, 5, 6, 8, 10, 12, and 15 cm used for depth dependencies considerations (Fig. S.3.2).

All evaluations were performed either on simple water geometry (presented in Figure 1) or patient CTs (head and abdomen region).

The simple geometry CT was virtually created by defining a box (6 cm × 6 cm × 6 cm) centered at different depths (depending on the scenario) within a virtual water phantom. In specific cases, additional structures simulating OARs or boost volumes were defined. This virtual water phantom was used to simulate a simplified setup, excluding the influence of real tissue inhomogeneities, providing a clearer understanding of interactions between the models.

For more complex evaluations representing clinical scenarios, two real patient CTs were used (head and neck region and abdomen). Both CTs were acquired with clinically used protocols for patients treated in our facility. The head and neck CT was used for calculation of the RBE‐weighted dose for a patient with a paranasal sinus tumor, where among all the structures contoured in the clinical workflow, only PTV and brainstem were visualized for the purpose of this study. The abdomen CT was taken for a patient with a sarcoma tumor, and for clarity of visualization, only CTV, PTV, and rectum were highlighted.

The physical dose distributions were computed with a pencil beam algorithm v4.4 and a 0.2 × 0.2 × 0.2 cm dose grid. For most considerations presented in this study, biological optimization was performed with the LEM model, and subsequently obtained physical doses were recomputed to MKM RBE‐weighted doses. The relation between dose distributions as well as corresponding RBE and LETd distributions were investigated in detail. All calculations were performed using the RayStation (V9a Ion and v11B, RaySearch Laboratories) treatment planning system. Evaluated doses per fraction were within our clinical range of 4.1–4.8 Gy RBE (LEM), and only for specific scenarios was a dose range between 2 and 15 Gy RBE (LEM) used. The Results section presents only a few selected examples; however, more detailed evaluations can be found in the Supplementary Material. Specific biological parameters used for calculations followed clinical standards as described in previous publications^13^ and are reported in Table 1.

### 2.3 Study outline

The study was divided into four sections covering various scenarios and their influence on the relation between both models and its impact on the treatment plan. The sections were defined as follows: (1) Dose dependencies, (2) Beam arrangement, (3) Gradients influenced by the OAR constraints, and (4) Spot distribution strategy. In the dose dependency section (1), emphasis was placed on the differences related to theoretical concepts behind both implemented models. Sections 2−4 are related to treatment planning parameters focusing on practical considerations often applied in clinical routine. The Supplementary Material includes further information about the range shifter (RaShi) influence, depth dependency, and Simultaneously Integrated (SIB) versus Sequential Boost (SEB) concept. Furthermore, additional examples with different doses per fraction are presented.

#### 2.3.1 Dose per fraction dependency

To consider the influence of the dose per fraction on the relation between the LEM and MKM model, a virtual geometry (described in Figure 1b) was used. For each of the considered dose levels (from 2 to 15 Gy RBE (LEM)), a single field homogeneous dose distribution within the planning volume box was generated, optimized with LEM model. Subsequently, each plan was recomputed with MKM model. Depth dose profile was generated and visualized as in Figure 1(a) (yellow line). Additionally, RBE depth profiles were generated for LEM and MKM and their respective dose per fraction levels.

#### 2.3.2 Beam arrangement

Several plans with various beam combinations were generated in the head and neck anatomy for the PTV receiving 4.8 Gy RBE per fraction in nine fractions (LEM optimized), subsequently they were recomputed with MKM model. Additionally, for each scenario corresponding LETd distributions were generated. Considered horizontal beam scenarios were defined as follows: one lateral beam (with table rotation 180°), two beams (180°+ 200°, 180°+230°, 180°+250°, 180°+270°, 180°+290°, 180°+310°, 180°+340°, 180°+360°), three beams (180°+270°+360°). Additionally, in the Supplementary Material similar considerations can be found for the virtual phantom (set up as defined in Figure 1b) with the prescribed dose 9 × 4.3 Gy RBE (LEM) and for head and neck geometry with the prescribed dose of 9 × 4.1 Gy RBE (LEM).

#### 2.3.3 Gradients influenced by the OAR constraints

LEM/MKM relationship was also tested in the presence of various gradients. To analyze that, a geometry in Figure 1(c) was chosen. In addition to the standard planning volume box, an OAR volume was placed distally, and various scenarios of OAR dose sparing were considered.

For each of the three evaluated scenarios, several plans were generated, each covered the target with the same LEM optimized dose—70 Gy RBE (LEM) (4.38, 5.00, 5.83, and 7.00 Gy RBE (LEM)/fraction), but the magnitude of OAR sparing differed. In scenario (a), OAR was spared to D1% < 30 Gy RBE (LEM), creating a very high gradient between PTV and OAR. In scenario (b), medium gradients were generated, by sparing OAR to D1 % < 45 Gy RBE (LEM). And finally scenario (c) represents low gradients, where the OAR was spared the least (D1 % < 60 Gy RBE (LEM)). Similarly, to other examples, each LEM optimized plan was recalculated to MKM doses, and depth dose profiles were generated as described in Figure 1(a).

#### 2.3.4 Spot distribution strategy

To evaluate the impact of spot distribution on the relation between the models, two strategies were considered. Two comparable plans, with the same beam configuration (two opposed beams), identical target coverage, and OARs sparing, all optimized using the LEM model. The only difference lay in the distribution of the spots within the plan.

- Constraint‐based scenario: spots from both beams could be placed freely within the target and located closely to critical organs. As a result, both beams were contributing to the entire target volume, without any restrictions. The desired dose distribution was driven purely by the set of constraints used during the optimization process.
- Blocking scenario: spots in each beam were physically blocked (use of custom blocking structures) to avoid placing spots in the critical OAR (brainstem). Since the brainstem was located centrally, blocking structures prevented beams' contribution to the contralateral part of the target. For this analysis, the head and neck case was chosen. Both LEM optimized plans were recomputed to MKM doses, and additionally LETd distributions were generated.

The Supplementary Material includes the example of the sarcoma patient, where three beams (two opposed lateral and posterior) were applied. The prescribed dose to the PTV1 was 9 × 4.8 Gy RBE (LEM) and the rectum was the OAR, for which spot placement was restricted.

## 3 RESULTS

### 3.1 Dose per fraction dependency

**FIGURE 2:**
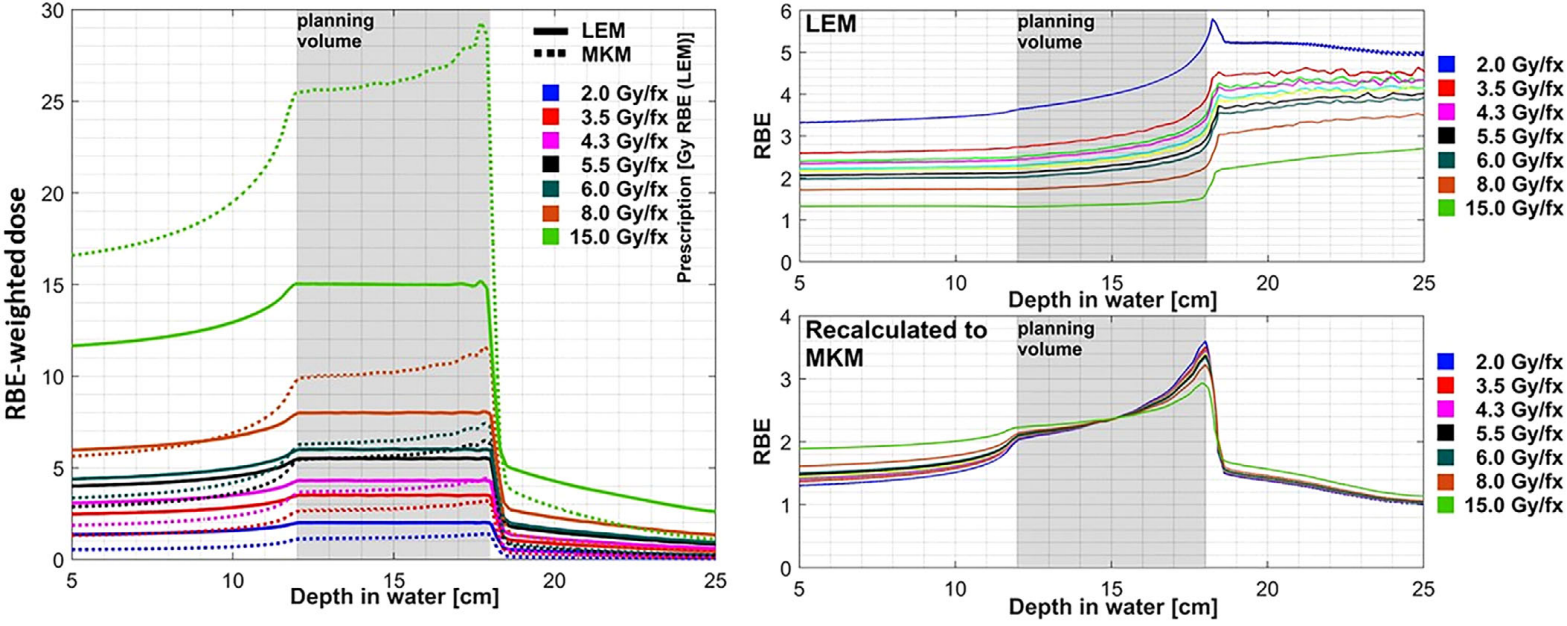
Dose line profiles along the beam direction through the target were evaluated for various LEM prescriptions and their respective MKM recalculations (left), behavior of RBE for LEM (top right), and for the recalculated MKM (bottom right).

Figure 2 presents the difference of the RBE‐weighted dose depending on the RBE model as a function of dose per fraction. For lower fraction dose (<3.8 Gy RBE (LEM)), the MKM RBE‐weighted dose is lower compared to the LEM RBE‐weighted doses in all regions (entrance plateau, proximal, mid and distal SOBP as well as fragmentation tail). Starting with approximately 4 Gy RBE (LEM) per fraction, both curves start to intersect. For even higher doses per fraction, MKM weighted doses are higher than LEM‐weighted doses. Figure 2(b, c) show corresponding RBE distributions as function of dose per fraction for LEM and recalculated MKM model, respectively. It can be clearly seen that RBE for LEM depends heavily on dose per fraction, whereas for MKM it varies minimally.

### 3.2 Beam arrangement

**FIGURE 3:**
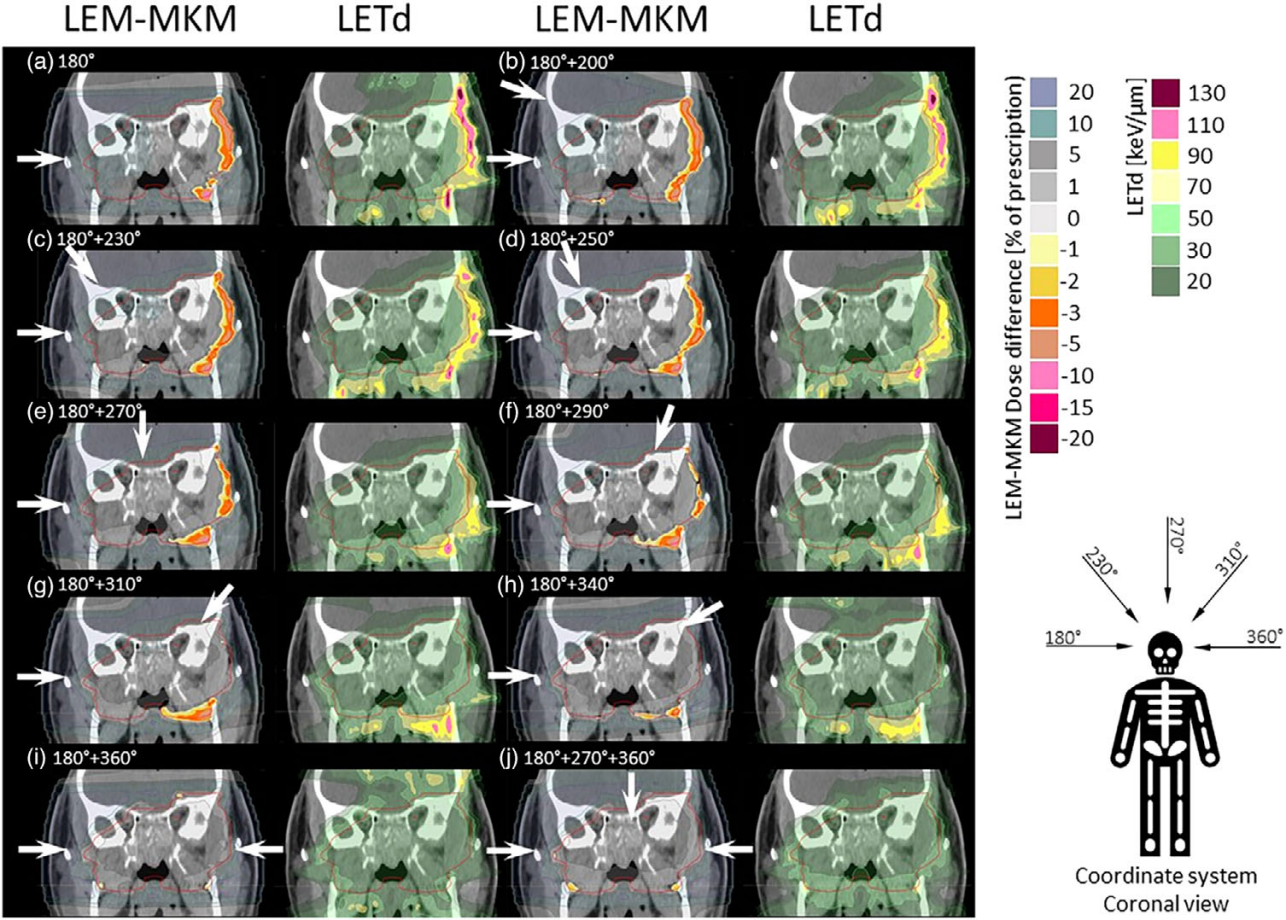
Impact of beam arrangements on the difference between LEM and MKM (left) and the LETd distribution (right). Red contours mark the planning target volume,which was planned with 9 × 4.8 Gy RBE (LEM). It can be observed that by adding another beam with increased spacing, the homogeneity between  LEM and MKM as well as LETd redistribution varies.

Figure 3 represents dose difference (left) between LEM optimized dose and recomputed MKM dose as well as LETd distribution (right) in the patient for a dose per fraction of 4.8 Gy RBE (LEM). Similarly to the previous example (Figure 2), for a single beam approach, it is expected to see the increase of the MKM recomputed doses in the distal edge of the SOBP (yellow to pink color scale). This area of the increased MKM corresponds to the increased LETd values (yellow to pink color scale). This unfavorable RBE and LETd distribution can be reduced by the application of an additional field. However, as it can be observed from Figure 3, 30 or 40 degrees field spacing (typically used in the clinical plans) is still resulting in significant regions where the MKM to LEM ratio is high at the distal part of the target and beyond. Moreover, the uncertainties in their estimations of the biological dose in this region may have severe consequences on the OARs located in the distal part of the beams. A homogenous LEM RBE‐weighted dose corresponds to a lower MKM RBE‐weighted dose in most of the volume, the areas with paradoxical MKM hot spot might have unwanted clinical consequences. With larger beam spacing those areas are reduced and almost removed completely for opposed beams (same with the three beams scenario). Observed results can be explained by the fact that high‐LET and high‐MKM regions (caused by end of range effect), are now redistributed within the tumor to opposite directions.

The described beam arrangement evaluation was repeated on the anatomical CT with the decreased dose per fraction (4.1 Gy RBE) and on the simple virtual phantom with 4.3 Gy RBE per fraction (Supplementary material). The conclusions for all considered scenarios were very consistent, but with higher dose per fraction, magnitude of observed changes increased.

### 3.3 Gradients influenced by the OAR constraints

**FIGURE 4:**
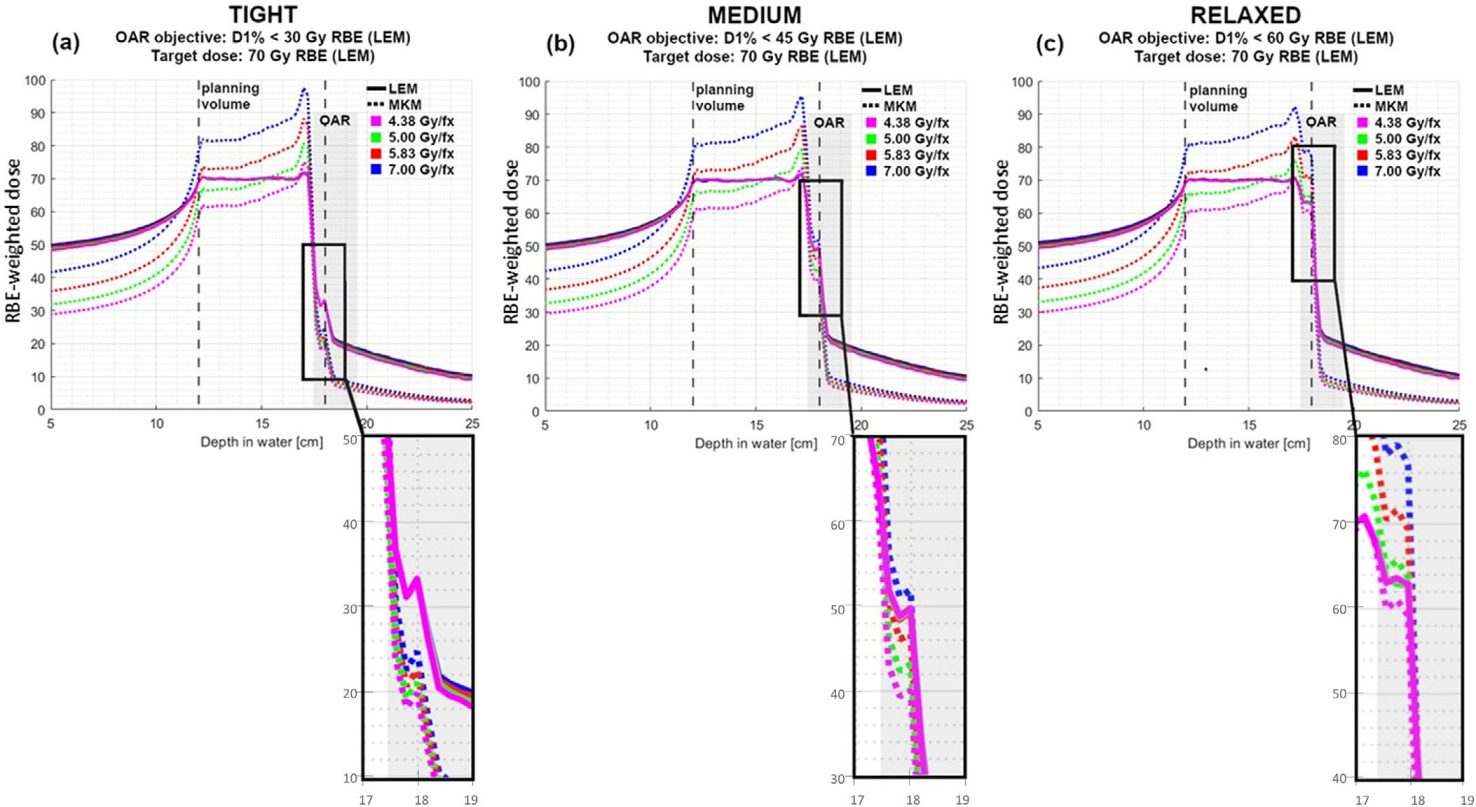
Distal gradient dependence of RBE models for an OAR overlapping with the planning volume. The OAR is assigned to different optimization objectives: (a) D1% < 30 Gy RBE (LEM) creating high gradient between PTV and OAR, (b) D1% < 45 Gy RBE (LEM), medium gradient between PTV and OAR, and (c) D1% < 60 Gy RBE (LEM) with very low gradient between PTV and OAR. Total target dose was for all fractionations and OAR settings equivalent to 70 Gy RBE (LEM). In the shown zoom‐in sections, it is evident that dose dependence for the MKM recalculation is increased if the OAR dose is comparably high, that is, in the case of relaxed constraints.

Figure 4 illustrates how gradients, influenced by the OAR doses (located distally to the target volume), will heavily depend on the model, its sensitivity to dose per fraction, and the total dose. In this scenario not only does the dose per fraction prescribed to the target matter, but also the degree of OAR sparing, and consequently, the dose gradients between those two volumes. While the MKM dose distribution (dotted lines) throughout the target seems to be very consistent across all three scenarios, the dose in the OAR varies significantly. Zoomed‐in regions of the graphs show how the RBE‐weighted doses differ between LEM and MKM models, depending on the magnitude of the OAR sparing and, therefore, the gradient between PTV and OAR. For scenarios with a very high gradient and significant OAR sparing, the MKM doses, after recalculation, are lower compared to LEM in the OAR region. Conversely, for scenarios with low gradients with mild OAR sparing, the situation is reversed, resulting in higher MKM doses after recalculation. This finding reflects the sensitivity of the relationship between the models, especially at the end of the particle range. This is particularly concerning from a treatment planning perspective, as adaptations of OAR dose constraints between the LEM and MKM model may be influenced by this end‐of‐range effect. In such situations, these translations cannot be trusted.

### 3.4 Spot distribution strategy

**FIGURE 5:**
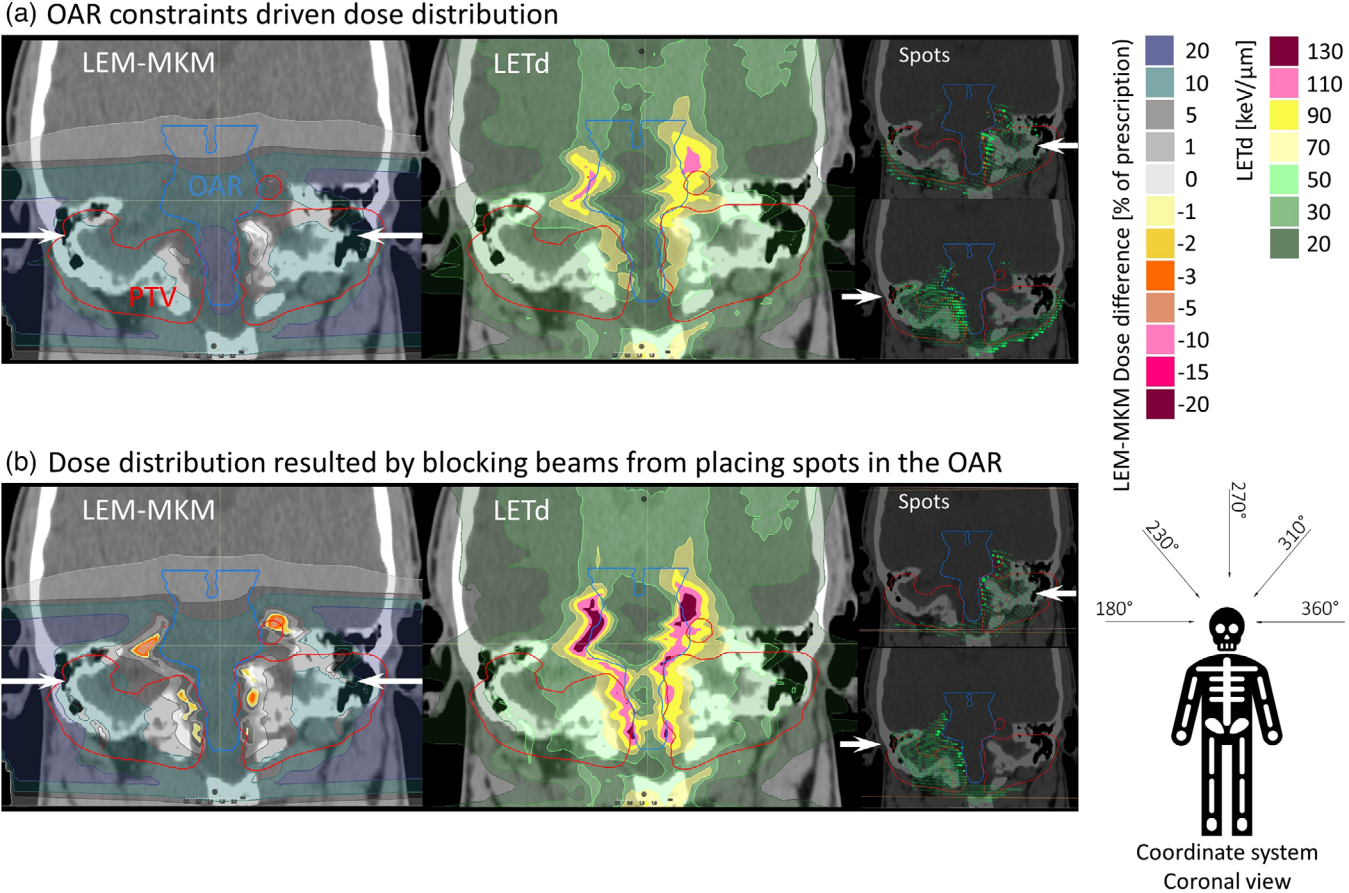
LEM‐MKM difference (left) and LETd distribution (middle) for two different strategies of spot distribution. On the right, a spot distribution from each field separately is shown. (a) OAR constraint‐driven dose distribution, where two opposed beams are allowed to place spots freely in the whole target volume. (b) OAR blocking strategy where both opposed beams are not allowed to place spots in the OAR (brainstem), resulting in the left beam contributing and placing spots only to the left part of the volume, and the right beam contributing and placing spots only to the right part of the volume. The OAR which is in the middle is not allowed to have any spots passing through, and essentially blocking them from stopping and contributing to the contralateral part of the target volume. White arrows represent beam directions. Both plans were optimized to achieve comparable RBE‐weighted dose distribution (LEM) in the target and sparing of the OARs.

Another example, where two plans with similar dose distribution (optimized with LEM), would result in different MKM dose distribution after recalculation as well as different LETd distribution is presented in Figure 5. The spot distribution strategy in the target and therefore beams’ contribution in the whole plan have major impact on conversion between LEM and MKM model. Figure 5(a) represents scenario, where spots from both beams are allowed to be placed freely, contributing to the entire target volume. In Figure 5(b), an alternative spot distribution strategy is presented, occasionally used in clinical practice, where spots are blocked from entering an organ‐at‐risk (e.g., brainstem), preventing any contribution to the contralateral part of the target. When comparing the dose differences between LEM and MKM for both strategies (left panel), noticeable variations in the presented dose distributions become evident, despite the initial similarity in LEM‐optimized plans. In the spot blocking strategy (Figure 5b), certain areas in front of the blocking structure exhibit higher MKM doses after recalculation compared to LEM (depicted in the orange color scale). These areas also show higher LETd values between scenarios, represented in the pink color scale. A similar example for the pelvis region is presented in the Supplementary material, yielding consistent findings.

## 4 DISCUSSION

The study aimed to summarize clinically relevant and practical differences between the two RBE models considered as standards in clinical dose computation for CIRT. Various factors were presented directly influencing the RBE‐weighted dose for both the LEM and MKM models. The translation between these models is not straightforward. The study demonstrated that in specific situations, seemingly similar LEM‐optimized plans can result in significantly different MKM and LETd distributions. This study highlights areas of concern that must be considered during the treatment planning process to ensure optimal CIRT treatment.

One of the most significant factors identified is the dependency of the models on the dose per fraction. This implies that the translations of prescribed doses or organ at risk (OAR) dose constraints, as presented in previous studies,^10^, ^11^, ^12^, ^13^, ^14^, ^15^, ^16^, ^17^, ^18^ are applicable only for the investigated dose per fraction. For increased fraction sizes, the accuracy of the translation would need to be studied separately and adjusted accordingly. Notably, in cases of fractionation above 4.8 Gy (RBE)/fx, where MKM consistently exceeds LEM, after recomputation, such conversion should be avoided. It is recommended to develop and validate distinct LEM‐to‐MKM translation models for various fractionation schemes to ensure that the translated constraints are valid for the specific dose range.

Furthermore, the strategies employed for generating a treatment plan have proven to significantly influence the validity of RBE‐weighted dose translation between the models.

Similarly, as for the physical range uncertainty, particle's distal beam edge is highly sensitive, and this area is where the most significant variations between models are expected. Opting to avoid dose deposition close to the OARs situated at the distal part of the beam and relying on lateral penumbra alone would be the simplest way to mitigate such uncertainties in the translation between the models. However, for the very complex clinical scenarios, it is not always possible to select a beam that does not partially stop in front of the critical OAR. Most of the CIRT‐centers lack a carbon gantry making the choice of optimal beam arrangement even more challenging. Additional degrees of freedom can be achieved only through several immobilizations of the patient in desired positions, but often, these mentioned limitations cannot be completely overcome.

Beyond beam directions, the strategy for spot distribution and gradients influenced by OAR sparing also contributes to the magnitude of distal edge uncertainty. This, of course, is driven by the dose per fraction (additional examples in Supplementary material). Moreover, by imposing hard constraints on a specific OAR (thus reducing the fraction dose in this OAR), the dose dependency of LEM to MKM relation can be minimized. That indicates that dose specific translations between the models may also depend on the actual constraint to the OARs (if located distally to the target).

Finally, all presented examples indicate that the areas where increased MKM doses were observed after recomputation from LEM also corresponded to the areas of increased LETd values. This information is particularly important for centers where LETd evaluation is not available yet and serves as an educated guess for CIRT plan assessment. Simultaneously, the study justifies the necessity of implementing additional tools, such as LETd evaluation and LETd optimization, in commercial treatment planning systems. Clinical evidence strongly suggests that LET distribution plays a crucial role in the outcome of CIRT.^28^, ^30^ Furthermore, our simple recalculation of LETd revealed that optimizing solely on RBE‐weighted dose may lead to a suboptimal LETd distribution.^31^

The incorporation of such tools in the commercial systems would allow in the future to move beyond model discrepancies and focus directly on the inherent physical properties influencing the effectiveness of CIRT.

Reported LETd distributions are based on the pencil beam algorithm calculations that have the monochrome beam model implemented (Raystation 9A Ion), which might be a limitation of this study. A trichrome beam model for RBE‐weighted dose calculation in CIRT, proposed by Inaniwa,^32^ found to be modelling the biological dose more accurately by including the contribution of the secondary, low radiation quality particles that spread widely around the central beam axis. Recently, we benchmarked the monochrome model implemented in the TPS (used for this study) against the Geant4 simulations and the trichrome model (implemented in the newer version of RayStation 11B)^33^ and observed no unexpected deviation or computation errors, except in the lateral direction outside the target where the dose is low (magnitude of a few keV).

Based on the above considerations, the obvious question that arises is related to the need for using both models during treatment planning and the practicability of LEM/MKM translations. The most valid argument for the importance of understanding both models is the fact that it is not possible to determine which one is better. Each model has its weaknesses, and the user cannot be certain which one is more accurate or if both lack in incorporating some important aspects, like LET‐volume effects.^29^ Mein et al. also noted that, based on in vitro experiments, MKM appears to be closer to reality, but this cannot be taken for granted in the clinical situation.^34^ Moreover, neither LEM nor MKM can be seen as ground truth for estimating biological effects within the patient as both are somehow based on in vitro data, they are not able to fully consider aspects occurring and influencing treatment success within the patient, for example, volume effects or variations in radiosensitivity between different cell types. Nevertheless, both models are successfully used in the CIRT over the years, achieving satisfactory clinical outcomes.

## 5 CONCLUSIONS

The study has compiled numerous practical treatment planning examples and synthesized clinical, experience‐based knowledge about the models. This summary equips users with the ability to apply recently published translation models for escalating dose constraints for specific OARs with increased awareness and confidence.

By introducing minor adjustments to the planning procedure based on the presented aspects, it becomes feasible to enhance the RBE and LETd distribution, potentially improving the clinical outcomes of CIRT. Understanding the relation between RBE models is particularly crucial, as not every commercial TPS has incorporated tools to evaluate both LEM and MKM models on a single platform, and even fewer have enabled LETd evaluation.

Building on the findings of this study and a prior publication on the MedAustron validation of translated constraints,^13^ our clinical workflow has been updated. Presently, two sets of OAR dose constraints are defined by the radiation oncologist: LEM and MKM constraints. Consequently, each clinical plan undergoes recomputation to MKM, and dose constraints for both models must be fulfilled. However, in scenarios where high dose regions are situated in close proximity to critical OARs, additional planning strategies often are employed. This may include an increased number of fields (particularly contralateral when possible), multiple immobilizations in different positions or planning organs at risk (PRV) margins.

## AUTHOR CONTRIBUTIONS

Joanna Góra (Physicist)—Idea generation, conceptualization, and study design. Carrying out certain parts of the study. Writing the first draft, editing, and preparing for submission. Sarah Grosshagauer (Student)—Conducting the majority of evaluations. Assisting in preparing the first draft. Creating the majority of the figures. Piero Fossati (Radiation Oncologist)—Brainstorming ideas, providing relevant input and evaluation from the medical/clinical perspective. Advising on the theoretical and practical implementation of models. Revising and editing the final paper version. Marta Mumot (Physicist)—Brainstorming technical ideas. Conducting parts of the evaluation related to SIB and SEB. Revising the final paper version. Markus Stock (Head of the Department)—Revising and editing several versions of the article. Providing technical input and contributing to writing and styling decisions. Mansure Schafasand (Physicist)—Assisting in the technical implementation of the models. Contributing to the evaluation of the LETd aspects of the study. Managing medical software. Antonio Carlino (Physicist)—Reviewing several versions of the manuscript. Offering scientific and technical insights.

## CONFLICT OF INTEREST STATEMENT

None.

## Supporting information

Supporting Information

## Data Availability

The data that support the findings of this study are available from the corresponding author upon reasonable request.
