# Supplementary material for: The sensitivity of radiobiological models in carbon ion radiotherapy (CIRT) and its consequences on the clinical treatment plan: Differences between LEM and MKM models
Source: J Appl Clin Med Phys. 2024 Mar 4;25(7):e14321. doi: 10.1002/acm2.14321 (PMC11244672; doi:10.1002/acm2.14321)
Supplement: Supplementary file 1 — Supporting Information [file ACM2-25-e14321-s001.pdf]

Supplementary material

S.1 Theoretical considerations - Model dependencies

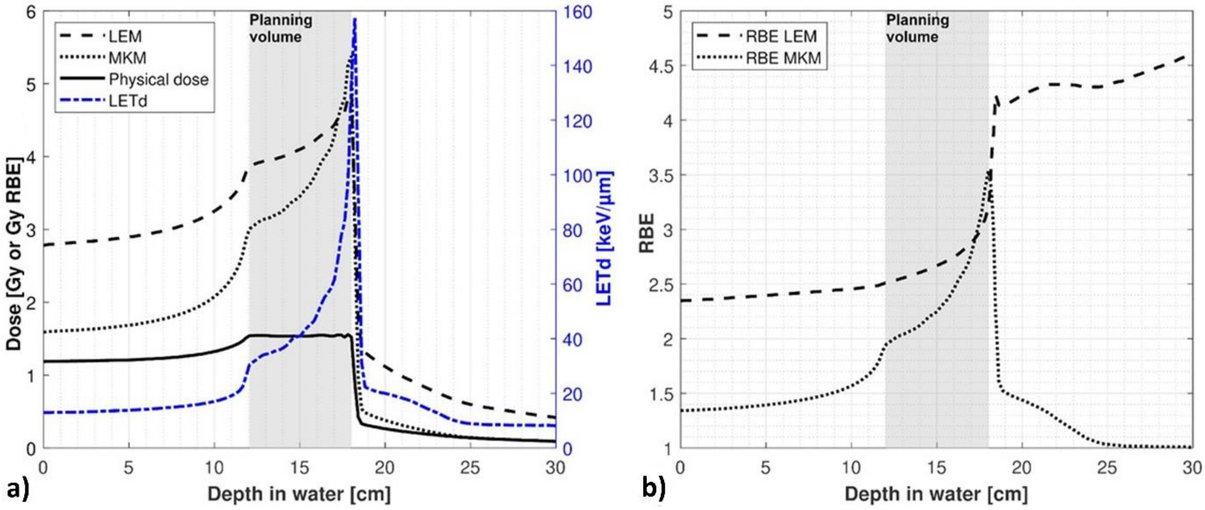

Figure S.1 displays RBE weighted doses calculated using both models, along with the LET distribution for a constant physical dose in a 6 cm width SOBP, as shown in (a), and the corresponding RBE, depicted in (b). The plan generated was optimized to achieve a D50% of 4.1 Gy RBE (LEM). The LETd dependence of MKM is clearly observable, whereas LEM estimates a significantly higher RBE in the initial plateau and the fragmentation tail.

The mathematical implementation of the LEM and MKM models in the TPS was previously reported and was outside the scope of this study. However, to provide a comprehensive overview, **Figure S.1a** illustrates the distinction between the two models. An example of a homogeneous physical dose profile (solid black line) is presented alongside its corresponding RBE-weighted dose distributions for LEM (dashed black line) and MKM (dotted black line). This is demonstrated on the optimized homogeneous physical dose, based on the prescribed LEM RBE dose of 4.1 Gy RBE. As expected from the theoretical definition, the LEM RBE-weighted dose is higher than the MKM RBE-weighted dose in the plateau region as well as throughout the majority of the SOBP and the tail region. Towards the end of the SOBP, divergent behavior is evident. In both models, the RBE-weighted dose at the end of the range

increases, albeit with dissimilar maxima, and notably, the MKM dose exceeds that of LEM. **Figure S.1b** illustrates the predicted RBE for LEM and MKM in the given scenario. The LEM RBE prediction tends to be significantly overestimated in the region of the fragmentation tail. Conversely, the MKM RBE prediction aligns in shape with the LETd distribution (as depicted by the blue line in **Figure S.1a**). The relation between both models is not linear; MKM is directly related to the LET distribution.

S.2 Beam arrangement

S.2.1 Simple geometry

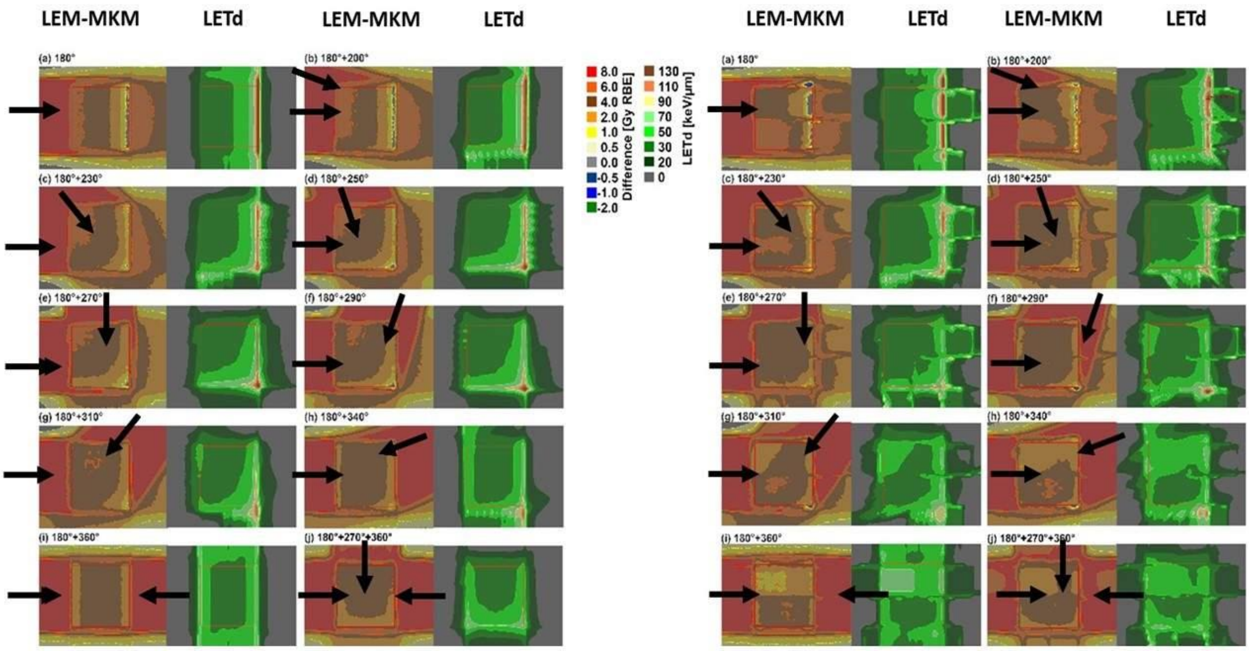

Figure S.2.1 illustrates the impact of beam arrangements on the difference between LEM and MKM, as well as the LETd distribution, for both homogeneous (left) and inhomogeneous (right) box configurations, with additional implementation of soft tissue material. Red contours delineate the planning target volume, which was planned with 9 × 4.1 Gy RBE (LEM). It can be observed that by adding another beam with increased spacing, the homogeneity in terms of difference, as well as LETd, is improved. Black arrows indicate beam directions

Figure **S.2.1 a,b** presents beam arrangement considerations, similarly as in chapter 2, in the virtual, simple box geometry.

S.2.2 Anatomical example (4.1 Gy RBE/fx)

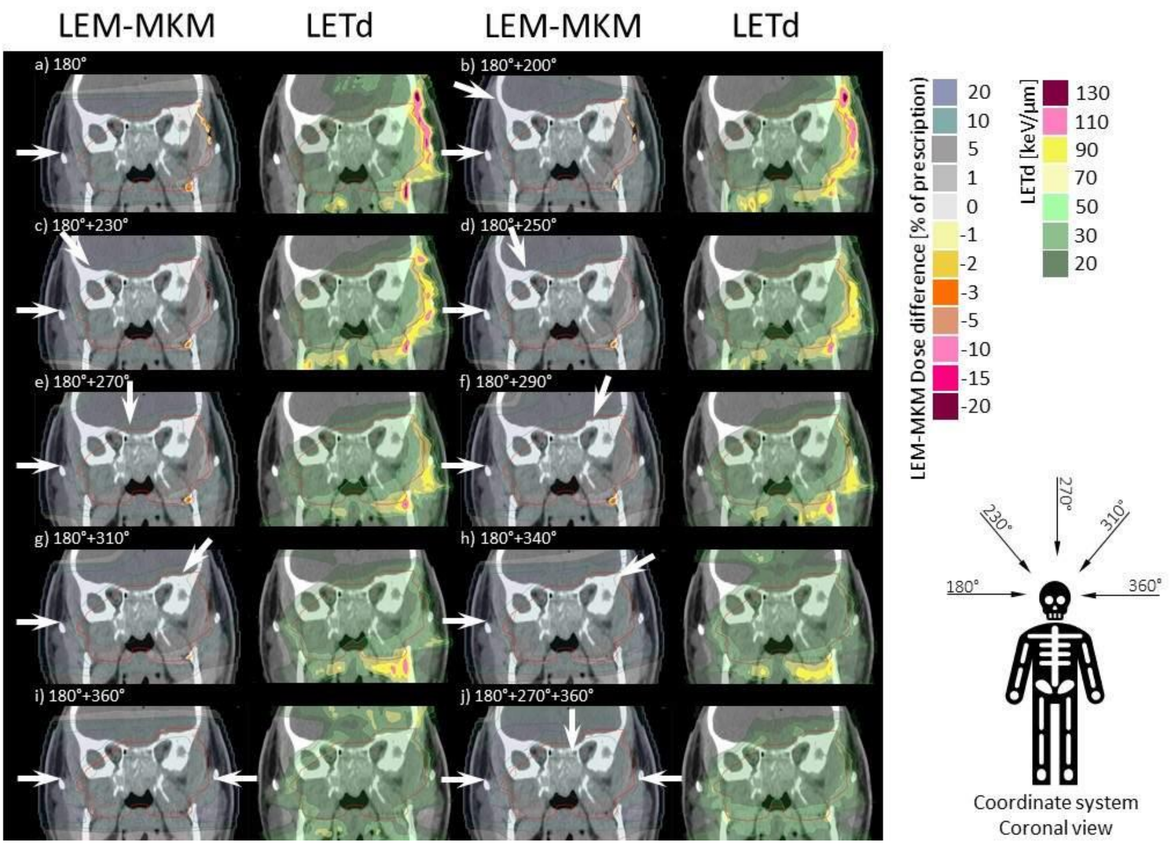

Figure S.2.2 illustrates the impact of beam arrangements on the difference between LEM and MKM, as well as the LETd distribution. Red contours delineate the planning target volume, which was planned with 9 × 4.1 Gy RBE (LEM). It can be observed that by adding another beam with increased spacing, the homogeneity in terms of both difference and LETd is improved.

Figure S.2.2 presents the same concept as **Figure 3**, except that this example was generated assuming a dose of 4.1 Gy RBE per fraction. As discussed earlier, it is expected that the extent of areas showing increased MKM after recomputation from LEM will depend on the dose per fraction. Therefore, the values of higher MKM, as well as LET, are less pronounced in **Figure S2.2** compared to **Figure 3**. Nonetheless, similar to **Figure 3**, it appears that two opposed beams provide the most optimal solution in terms of consistent RBE-weighted dose and LET distribution.

S.3 RaShi vs. noRaShi considerations

This section explores whether RaShi, implemented in the pencil beam algorithm for dose computation in shallow targets, influences the relationship between LEM and MKM RBE-weighted doses.

S3.1. LEM/MKM relation between Rashi and non-Rashi plan

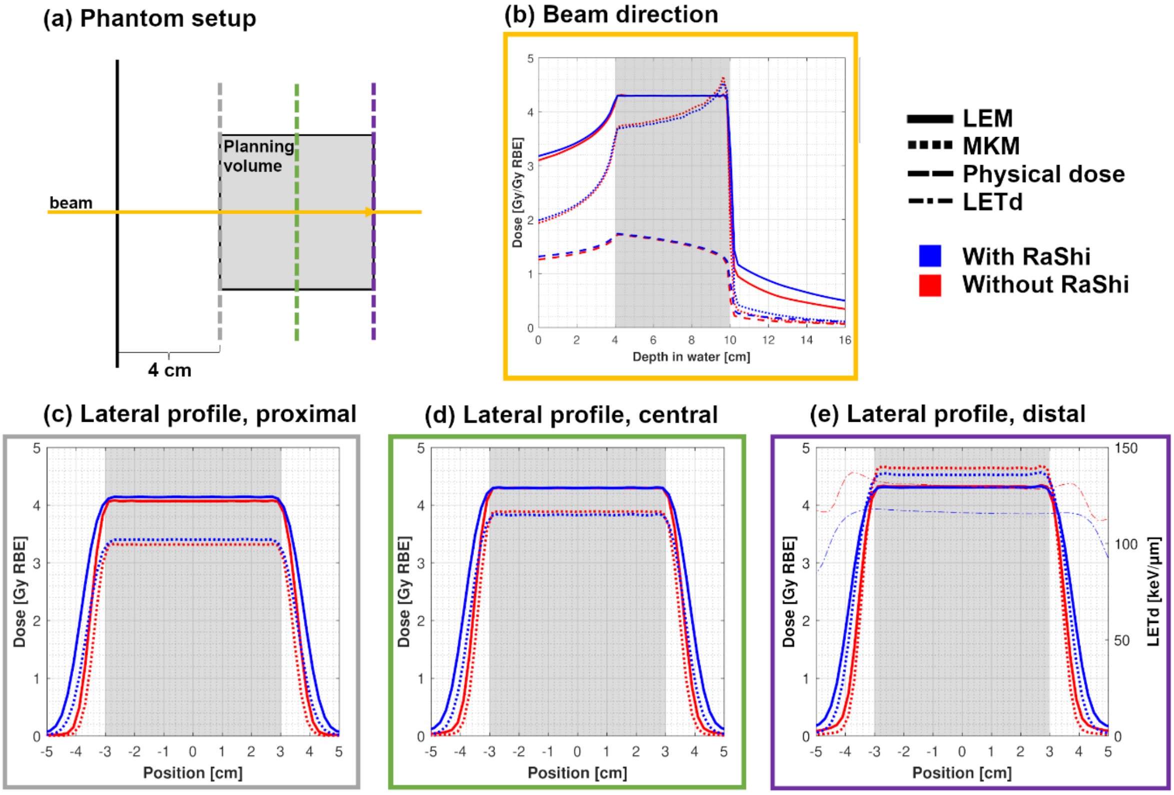

Figure S.3.1: Relationship between LEM and MKM models for plans optimized with and without Rashi. (a) Virtual water phantom setup, with yellow horizontal lines and grey, green, and purple vertical lines indicating the positions of corresponding profiles. (b) Depth dose profile. (c) Lateral profile in the proximal area of the target (grey). (d) Lateral profile in the central part of the target (green). (e) Lateral profile in the distal part of the target (purple). In each graph, dashed lines represent physical doses, solid lines represent LEM doses, and dotted lines represent MKM recomputed doses. Graphs in blue correspond to plans with Rashi beam, while those in red were computed without the use of Rashi

Figure S.3.1 illustrates the physical, LEM RBE-weighted, and MKM RBE-weighted dose in water with and without the use of Rashi in the computation (blue and red color, respectively). The depth dose profile of the physical dose for both Rashi and non-Rashi plans is almost

identical, with the non-Rashi plan showing slightly lower doses in the plateau and tail regions. This outcome is unsurprising, as the influence of Rashi is directly related to the physical dose, and the observed differences are primarily due to the fact that Rashi beams require higher energies to cover the same target.

S.3.2 Depth and dose per fraction dependance

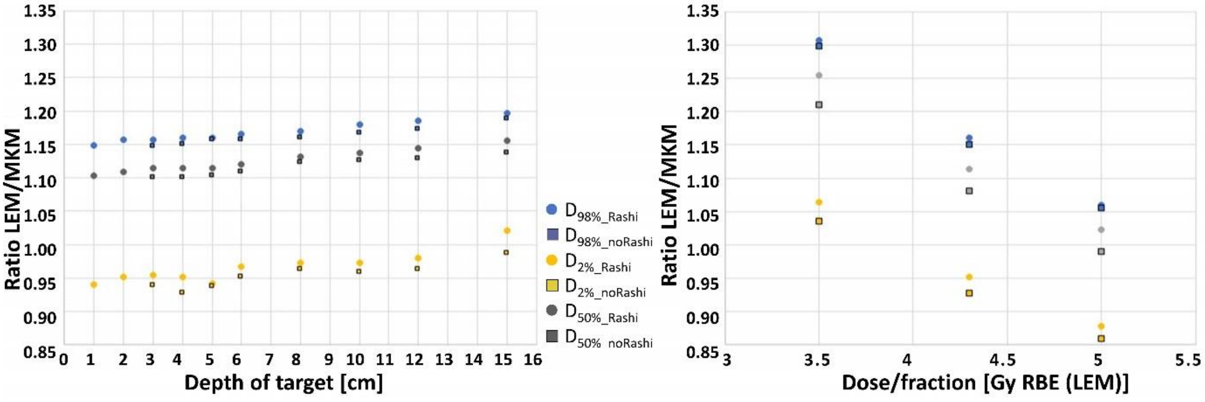

Figure S.3.2: (a) LEM/MKM ratio for different depths. Circles represent Rashi values, squares represent noRashi values. In blue: D98% of the prescribed dose, in yellow: D2% near max doses, and in green: median dose in the target. (b) LEM/MKM ratio depending on the fraction dose for Rashi vs non-Rashi beam. Circles represent Rashi beam, squares represent non-Rashi beam.

In **Figure S.3.2a**, we present the results of several plans optimized for the same dose, fraction size, and target volume at different depths ranging from 1 to 15 cm in water. These calculations were performed for a single beam with and without range shifter (Rashi). The relationship between the LEM and MKM models is depicted as the LEM/MKM ratio for near-min doses (D98%), median doses (D50%), and near-max doses (D2%). Notably, the ratio between LEM and MKM increases with depth across all presented parameters. Furthermore, the LEM/MKM ratio for Rashi plans consistently exceeds that of non-Rashi plans, although it remains constant.

In **Figure S.3.2b**, we explore the LEM/MKM ratio for three different fraction sizes (3.5 Gy RBE, 4.5 Gy RBE, and 5 Gy RBE) and its relation between Rashi and non-Rashi beams for specific

dose parameters (D98%, D50%, and D2%). Despite significant variations in the absolute LEM/MKM ratio values depending on the fraction size, our results indicate that the relative value of the ratios between Rashi and non-Rashi plans remains consistent for each selected parameter.

S.4 Simultaneously integrated boost (SIB) vs sequentially delivered boost (SEB)

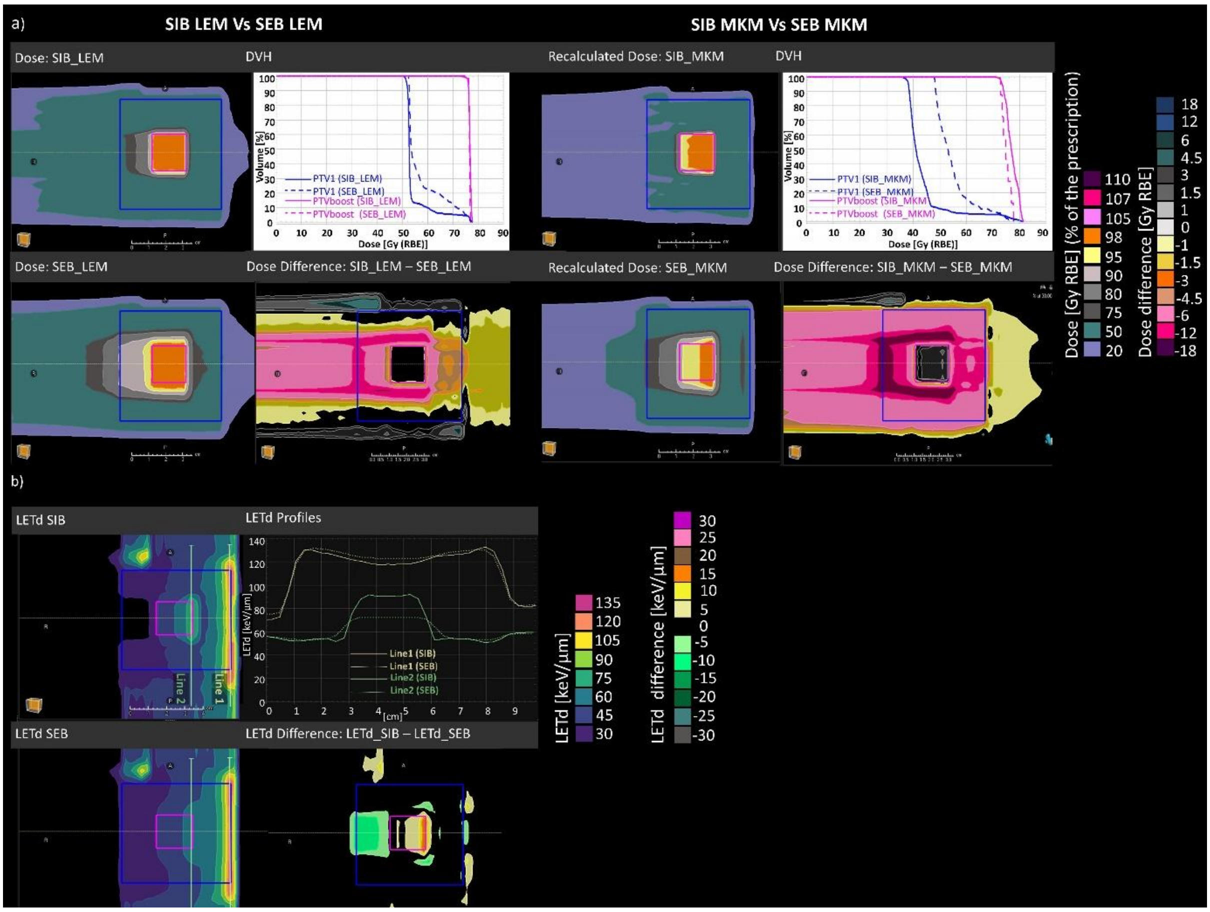

Figure S.4 Comparison between SIB vs SEB strategy and its influence on the recomputed MKM doses. Large target was assigned to have 76.8 Gy RBE (LEM), while smaller target supposed to get 52.8 Gy RBE (LEM) (16 fx for SIB, 11fx+5fx for SEB). Single lateral field has been chosen. A) presents the difference between SIB and SEB plan optimized with LEM model (left) and its recalculation to MKM (right). B) Corresponding LETd variations for both planning strategies. White arrow indicated the field direction.

Figure S.4 depicts a conceptual comparison of the benefits and drawbacks associated with the Simultaneous Integrated Boost (SIB) versus Sequentially Executed Boost (SEB) approach,

as well as their influence on the relationship between the models. Notably, the clinical correctness and biological sense were not taken into account for this example.

Both SIB and SEB plans were optimized with the LEM model (1 lateral field) to achieve comparable coverage of both targets, PTV1 and PTVboost. Initially, the conformity of the plan, especially for the larger target (PTV1), appeared to be better for SIB (**Figure S.4a, left**). However, after recomputation to MKM, it became evident that the SIB MKM coverage for the larger target would be considerably smaller than for SEB MKM (**Figure S.4a, right**).

Regarding the LETd distribution, both techniques exhibited similar values for the larger target. However, the smaller target displayed considerably higher LETd for the SIB technique (**Figure S.4b**)

S.5 Spot distribution strategy (sarcoma example)

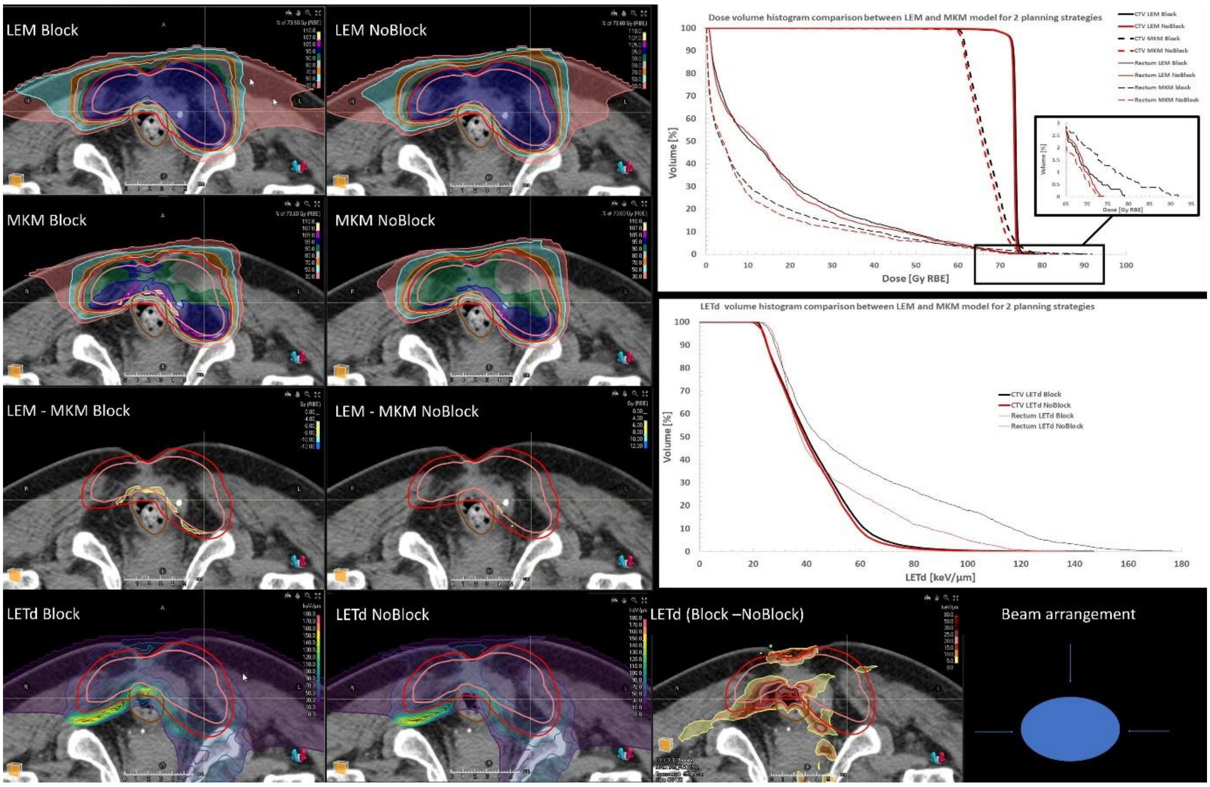

Figure S.5: Example of both planning strategies for the same sarcoma patient treated with 4.6 Gy RBE (LEM) per fraction (in 16 fractions), with the same beam arrangement. Blocking strategy (left): spots from both lateral fields were not allowed to enter the rectum, while the vertical beam was not blocked. NoBlock strategy (middle): each of the three beams was allowed to place spots freely, resulting in some spots entering the rectum. The top row represents doses optimized with the LEM model; both plans were comparable in terms of coverage and organ-at-risk (OAR) sparing. The second row represents LEM-optimized plans recomputed with the MKM model. The third row shows the dose difference between LEM and MKM (only values where MKM is higher than LEM are displayed for clarity reasons). The fourth row represents the LETd distribution of both planning approaches: LETd for the Block plan, LETd for the NoBlock plan, and LETd difference between the Block and NoBlock plans, respectively. Top left: DVH comparison between plans for CTV and Rectum (zoomed-in section presents max doses for the rectum). Middle left: LETd histogram for CTV and rectum.

Similarly to the concept from Chapter 4, **Figure S.5** presents two scenarios for the sarcoma patient: on the left, each of the three beams was blocked from placing spots in the rectum (brown structure), while in the middle, a dosimetrically equivalent plan (in terms of LEM dose distribution) can be seen, where each beam was allowed to place spots freely, thus contributing to the entire target volume. In the third row, it can be seen very clearly the region where the MKM RBE-weighted dose is much higher for the blocked scenario compared to the unblocked scenario. Similarly to the head and neck scenario, higher MKM regions corresponded to the higher LETd regions (fourth row).
